# Remifentanil at induction of general anesthesia for cesarean section: Double blind,randomized clinical trial

**Published:** 2013-06-30

**Authors:** Shekoufeh Behdad, Vida Ayatollahi, Hamid Harrazi, Naderali Nazemian, Najmeh Heiranizadeh, Behnam Baghianimoghadam

**Affiliations:** a Shahid Sadoughi Hospital, Shahid Sadoughi University of Medical Sciences, Yazd, Iran. E-mail: ayatollahividatryuior@yahoo.com; b Shohaday-e-kargar Hospital.Yazd. Iran, E-mail: harrazihamidftryui@yahoo.com

**Keywords:** Remifentanil, Placebo, caesarean

## Abstract

**Introduction:**

Remifentanil, with its rapid activity onset and short duration of action, may be more effective than other opioids for providing hemodynamic stability during obstetric anesthesia. However, there is some evidence of adverse effects on neonatal respiratory function. We investigated maternal and fetal effects of Remifentanil during cesarean section surgery.

**Methods:**

Eighteen women with singleton term pregnancies, and physical class status of I or II as defined by the American Society of Anesthesia (ASA), who were undergoing general anesthesia for semi-elective cesarean section were randomized into two groups (40 in each group) that received either an intravenous bolus of 0.5 µg/kg Remifentanil or the same dose of saline as a placebo. Maternal hemodynamic variables and neonatal umbilical artery pH and Apgar score at first and fifth min were evaluated in both groups.

**Results:**

Systolic and diastolic blood pressure were significantly lower after tracheal intubation and skin incision in the Remifentanil group as compared with the control group (*p *<0.05). There were no significant differences regarding heart rate between groups at any time (*p *> 0.05). Apgar scores at first and fifth min were not significantly different among groups (*p*>0.05). No neonate required assisted ventilation or naloxan administration.

**Conclusion:**

Remifentanil may be a safe and effective drug for the induction of general anesthesia and surgical stimulation without subsequent neonatal depression.

## Abstract (es)

**Introducción:**

El Remifentanilo, con su rápido comienzo de actividad y corta duración de acción, puede ser más eficaz que otros opioides para proporcionar la estabilidad hemodinámica durante la anestesia obstétrica. Sin embargo, hay alguna evidencia de efectos adversos sobre la función respiratoria neonatal. Se investigaron los efectos maternos y fetales de Remifentanilo durante la cirugía cesárea.

**Métodos:**

Dieciocho mujeres con embarazo de feto uno y con clasificación Asa I o II (sociedad americana de anestesiología) y que fueron sometidas a anestesia general para ces´rea semi eletiva, se dividieron aleatoriamente en dos grupos (40 mujeres en cada grupo) que recibieron un bolo intravenoso de 0.5 mm/Kg de remifentanil o en el grupo control la misma dosis de solución salina como placebo. Se evaluaron las variables hemodinámicas maternas y pH de la arteria umbilical neonatal y la puntuación de Apgar al primer y quinto minuto en ambos grupos.

**Resultados:**

La presión arterial sistólica y diastólica fueron significativamente más bajos después de la intubación traqueal y la incisión de la piel en el grupo Remifentanilo en comparación con el grupo control (*p* <0.05). No hubo diferencias significativas en cuanto a la frecuencia cardíaca entre los grupos en cualquier tiempo (*p*> 0.05). Las puntuaciones de Apgar a los minutos primero y quinto no fueron significativamente diferentes entre los grupos (*p*> 0.05). No neonato requiere ventilación asistida o administración naloxan.

**Conclusión:**

El Remifentanilo puede ser un fármaco seguro y eficaz para la inducción de la anestesia general y la estimulación quirúrgica sin depresión neonatal posterior.

## Introduction

Cesarean section is a common obstetric surgery and the anesthetic technique selected by anesthesiologists for this kind of operation is very important. While regional anesthesia is the preferred method for cesarean section, in high risk obstetric conditions, such as maternal coagulopathy, cardiac and neurologic diseases and cases of urgent delivery, general anesthesia is indicated^1^. Opioid drugs are not usually given for induction of general anesthesia for cesarean section because of neonatal respiratory depression^2^ ^,^ ^3^; however, without opioid administration, patients are at risk of complications, such as increased heart rate and hypertension following laryngoscopy, tracheal intubation and skin incision. These neuroendocrine stressors can be life-threatening in mothers with cardiovascular diseases. In such cases it is necessary to consider a safe and effective analgesic agent for such situations.

The characteristics of Remifentanil make it a good choice for usage before delivery instead of other opioids. Remifentanil is a synthetic µ-specific agonist opioid with a rapid onset of action, a short duration to reach peak effect, a rapid clearance and quick fetal metabolism^2^. Also, it can be administered for prevention of unwanted maternal hemodynamic instability during induction of general anesthesia and provides cardiovascular stability in high-risk patients^4^ ^,^ ^5^. Remifentanil has been successfully used in cases of maternal cardiac diseases^6^ ^-^ ^9^ intracranial cysts^10^, and preeclampsia^11^.

Although previous studies reported newborn chest wall muscle rigidity for short periods^12^, as well as neonatal depression^13^ ^,^ ^14^ following Remifentanil administration, other studies suggest that low Apgar and neonatal depression may be dose-dependent and the use of a low dose of Remifentanil may be safe for newborns^15^ ^,^ ^16^.

In this study we investigated the maternal and neonatal effects of Remifentanil as compared with the administration of a saline solution during the induction of general anesthesia for cesarean delivery.

## Material and Methods

A double blind, randomized, placebo-controlled clinical trial was conducted from October 2009 to November 2010 at the Shahid Sadoughi Hospital, Yazd, Iran. After a review by the university institutional ethics committee, the study was registered in the Iranian registry of clinical trials (http://irct.ir) as IRCT201102252963N2. Forty patients were chosen for each group on the basis of similar studies with a power of 85% and α confidence level of 0.05. A minimal difference to be detected in heart rates was set at 10 beats.

Women with singleton, term pregnancy with a physical status of I or II as defined by the American Society of Anesthesia (ASA) were included in the study. Patients were selected from a pool of approximately 1,000 C/S candidates. All patients were candidates for elective cesarean section by general anesthesia because of contraindications for regional anesthesia. The indicators for induction of general anesthesia included: refusal of spinal anesthesia by patients, anticoagulation therapy, and previous spinal surgery.

**Figure 1:**
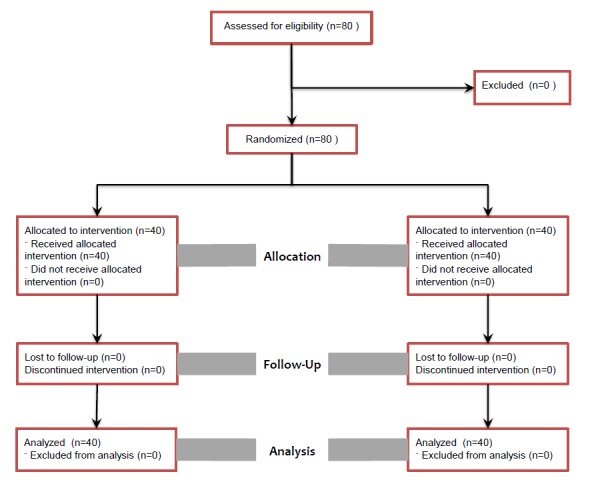
Flow of the study

Patients with preeclampsia, cardiovascular and neurologic diseases, hypertension, hepatic insufficiency, renal failure, thyroid dysfunction, drug or alcohol abuse history, known fetal abnormalities, and predicted difficulty with airway management were all excluded from the study. The patients were randomly divided into two groups: an experimental group with Remifentanil administration (n= 40) and a control group with normal saline injection (n= 40) (Fig 1). Random selection to each group was performed by drawing a piece of paper from a bag containing equal numbers for each method option. The randomization process was conducted by a biostatistician who was blinded to patient groups during the randomization and analysis processes. A written informed consent was obtained from patients before starting the procedure.

### Anesthetic management.

For premedication, 10 mg metoclopramide was given one hour before induction of the anesthesia. In the operating room, two IV lines were inserted and 1,000 mL lactating ringer solution infused before the induction of anesthesia.

Routine monitoring included non-invasive measurements of blood pressure, electrocardiography, pulse oximetry and capnography. Systolic blood pressure (SBP), diastolic blood pressure (DBP), mean arterial pressure (MAP) and heart rate (HR) were recorded before induction of anesthesia, immediately after laryngoscopy and tracheal intubation, immediately after skin incision and after delivery by using a BCI Advisor Vital Signs Monitor. Hemodynamic variables were measured by anesthetic technicians who were unaware of patient groups.

The patients were pre-oxygenated for three min before the induction of anesthesia while in a supine position. General anesthesia was induced by using a rapid sequence administration of sodium thiopental 5 mg/kg and succinylcholine 1.5 mg/kg. Following direct laryngoscopy and endotracheal intubation within 15 seconds, anesthesia was maintained with 0.7% isoflurane in a 50% nitrous oxide with 50% oxygen mixture. Isoflurane concentration was decreased to 0.5% after delivery and a dose of 0.5 mg/kg atracurium was administered as a muscle relaxant.

In the experimental group, a 0.5 µg/kg Remifentanil bolus was injected over 30 seconds immediately before induction of anesthesia. For the control group; the same volume of saline was infused as a placebo.

After delivery and clamping the umbilical cord, 1.5 µg/kg fentanyl was given to patients of both groups. Following extubation, postoperative analgesia was managed using a patient-controlled morphine injection pump.

After delivery, neonates were evaluated by a pediatrician who was blinded to the mother´s group. The Apgar scores at the first and fifth min were also determined. Additionally, the umbilical artery pH of the newborns was assessed by the use of the Gem 3000 Blood Gas Analyzer.

All registered data were digitalized for the SPSS(r) 15 software and were analyzed by T-test. *P*-value < 0.05 was considered as the level for statistical significance.

## Results

Demographic maternal, and fetal characteristics of the two groups are indicated and compared in Table 1. There was no significant difference found between the two groups studied, according to the demographic characteristics of participants. Maternal age, maternal weight, the duration of pregnancy, as well as the neonatal weight were not statistically different between the groups studied. Elective (non-emergent) cesarean section was performed for all women. The duration between uterine incision and delivery was found to be 51.53 ± 9.50 and 49.34 ± 9.20 in Remifentanil and control groups, respectively (*P* > 0.05).

**Table 1.**
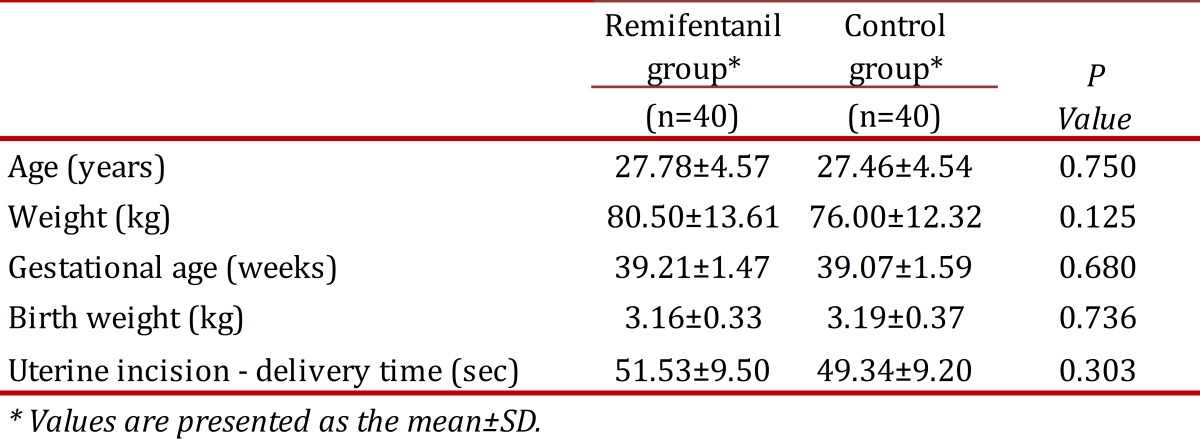
Maternal and neonatal characteristics in both groups.

No differences were found between the groups regarding systolic and diastolic blood pressure before induction of anesthesia and after delivery. Systolic and diastolic blood pressures were reduced significantly after tracheal intubation and skin incision in the Remifentanil group when compared with controls (*p *<0.05) (Table 2). There was no significant difference between the two groups regarding heart rate at any time (Fig 1). All neonates had Apgar scores above seven after the first minute. Assisted ventilation, tracheal intubation and naloxane administration were not necessary.

**Table 2.**
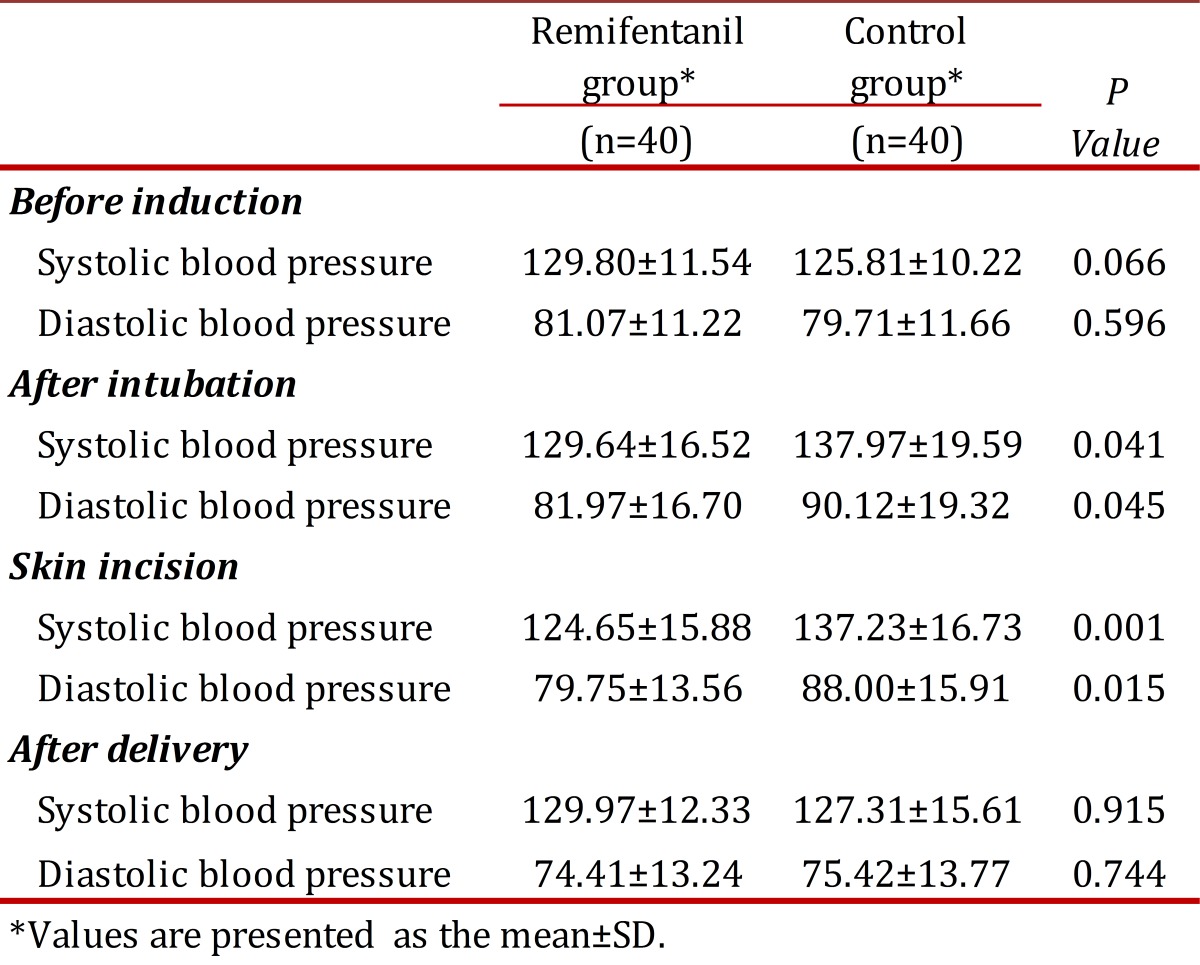
Blood pressure changes between induction and delivery in both groups.

The mean first-minute Apgar scores were 8.83 ± 0.79 and 8.87 ± 0.40 for the Remifentanil and control groups, respectively, with no statistical differences (*p* >0.05). Mean Apgar scores at the fifth min were 9.90 ± 0.48 and 9.97 ± 0.16 for the experimental and control groups, respectively, with no significant differences (*p* >0.05). Umbilical artery blood pH was measured as 7.37 ± 0.07 for the Remifentanil group and 7.35 ± 0.02 for the control group. Differences were not found to be significant (*p* > 0.05).

## Discussion

In cesarean section under general anesthesia, it is ideal to minimize maternal surgical stress and newborn depression.Based on the present study, significant reduction in systolic and diastolic blood pressures after laryngoscopy, intubation and skin incision was found among women in the Remifentanil group when compared with the control group. The results indicated that a single bolus of 0.5 µg/kg Remifentanil may prevent the hypertensive responses following laryngoscopy, tracheal intubation and skin incision.

The positive effect of Remifentanil on hemodynamic stability was observed in previous studies using different doses of Remifentanil. Van de Velde *et al* administered 0.5 µg/kg bolus of Remifentanil followed by continuous infusion of 0.2 µg/kg/min combined with propofol. A constant mean arterial pressure during anesthesia was reported^15^. Similarly, Ngan Kee *et al* used a bolus of 1 µg/kg Remifentanil before induction of anesthesia and found that patients in the placebo group had higher SBP and MAP when compared with the Remifentanil group^3^. In contrast, Draisci *et al* who used 0.5 µg/kg bolus of Remifentanil followed by a continuous infusion of 0.15 µg/kg/min did not find any significant differences in blood pressure between the Remifentanil and control groups^13^.

No significant differences between the two groups regarding SBP and DBP after delivery were found in our study. This may be explained by the prompt action and rapid metabolism of Remifentanil. The mean induction-to-delivery interval found in this study was 7.5 min. It is assumed that Remifentanil with its short half life^17^ did not decrease the blood pressures after delivery in the experimental group. Similarly, in the Draisci *et al* study^13^ no significant differences were found regarding the blood pressure at the end of surgery between the groups studied. However, Van de Velde *et al* found a significant difference in mean arterial pressure at the time of delivery between the groups studied^15^.

Our study was similar to that of the Draisci *et al* survey in that no significant difference was found in the heart rates between the studied groups^13^. In contrast, Ngan Kee *et al* ^3^ reported that the heart rate for the control group was higher than that found for the Remifentanil group. This difference may be due to the lower dose of Remifentanil used in our study when compared with the dose used in the Ngan Kee study. Management of the increased heart rate following laryngoscopy and intubation seems to require higher doses of anesthetic and analgesic drugs^18^.

In the current study, no differences were found between groups according to the mean Apgar scores at the first and fifth min. All neonates had Apgar scores higher than seven at the first minute and no neonate required naloxan administration or tracheal intubation. Given the Remifentanil dose used in our study, it appears safe for use with newborns. However, Ngan Kee claimed that even low doses of Remifentanil can cause neonatal respiratory depression. Despite giving naloxan to 10% of the neonates in the Remifentanil group, our study found that no differences were present between groups regarding the Apgar score^3^. There are, however, other reports concerning naloxan administration^10^ where apnea and generalized chest wall rigidity^12^ ^,^ ^19^ in neonates was found when Remifentanil was given during cesarean section.

Kan *et al* reported a high transplacental passage of Remifentanil, but only transient neonatal depression^2^. Van de Velde´s study reported that approximately 50% of infants showed a brief respiratory depression with the need for naloxan administration or assisted ventilation and that two of 13 neonates studied had Apgar scores less than seven at the fifth-minute evaluation^15^. This disparity may be due to the use of Remifentanil in combination with propofol as was done in the Van de Velde study. Draisci *et al* found a significant reduction in Apgar scores at the first and fifth min in the Remifentanil group; however, all Apgar scores were greater than eight at the fifth minute without requiring naloxan. Further, Draisci indicated that a high bolus dose and a lower infusion of Remifentanil may be useful for surgical stress management with minimal neonatal sedation^13^.

According to our study, by avoiding the use of Remifentanil infusion, neonatal depression may be decreased to minimum level because of the rapid metabolism of the single dose of Remifentanil. However, in the Van de Velde study, a single bolus dose of Remifentanil caused respiratory depression in two neonates that required naloxan administration^15^. It should be noticed that the single bolus dose of Remifentanil which was used in Van de Velde´s study was two times greater than the single bolus dose we used. By using a single bolus of 0.5 µg/kg Remifentanil in our study, no neonatal depression occurred and all neonates had Apgar scores greater than seven without naloxan being required.

Mean umbilical artery pH was greater than 7.35 in both groups of neonates. Since low umbilical artery pH reflects both respiratory and metabolic acidosis, it can be concluded that Remifentanil administration in the dose given in our study will possibly not cause respiratory and metabolic acidosis. Similar results were reported in other studies^13^ ^,^ ^15^. However, some neonates in these studies experienced fetal distress following Remifentanil administration to their mothers. In a cohort study, only 19% of the infants with low Apgar scores had severe acidosis at birth and 27% of the infants with acidosis had a low Apgar score at the fifth minute^20^.

## Conclusion:

According to our observations, the administration of a single bolus dose of Remifentanilin women undergoing caesarean under general anesthesia, may possibly reduce systolic and diastolic blood pressures, but not the heart rate. Remifentanil with its rapid onset of action and rapid metabolism can be recommended as a safe opioid with minimal newborn depression, especially when it is administered in a single bolus dose. It seems that further studies with larger sample sizes and different Remifentanil doses are required to evaluate the risk/benefit ratio for both mothers and neonates.
